# Chemical and Metabolic Aspects of Antimetabolite Toxins Produced by *Pseudomonas syringae* Pathovars

**DOI:** 10.3390/toxins3091089

**Published:** 2011-08-31

**Authors:** Eva Arrebola, Francisco M. Cazorla, Alejandro Perez-García, Antonio de Vicente

**Affiliations:** 1 Instituto de Hortofruticultura Subtropical y Mediterránea “La Mayora” (IHSM-UMA-CSIC), Estación Experimental La Mayora, Algarrobo-Costa, Málaga 29750, Spain; 2 Instituto de Hortofruticultura Subtropical y Mediterránea “La Mayora” (IHSM-UMA-CSIC), Departamento de Microbiología, Facultad de Ciencias, Universidad de Málaga, Unidad Asociada al CSIC, Campus de Teatinos, Málaga 29071, Spain; Email: cazorla@uma.es (F.M.C.); aperez@uma.es (A.P.-G.); adevicente@uma.es (A.V.)

**Keywords:** tabtoxin, phaseolotoxin, mangotoxin, virulence, arginine, ornithine, glutamine

## Abstract

*Pseudomonas syringae* is a phytopathogenic bacterium present in a wide variety of host plants where it causes diseases with economic impact. The symptoms produced by *Pseudomonas syringae* include chlorosis and necrosis of plant tissues, which are caused, in part, by antimetabolite toxins. This category of toxins, which includes tabtoxin, phaseolotoxin and mangotoxin, is produced by different pathovars of *Pseudomonas syringae*. These toxins are small peptidic molecules that target enzymes of amino acids’ biosynthetic pathways, inhibiting their activity and interfering in the general nitrogen metabolism. A general overview of the toxins’ chemistry, biosynthesis, activity, virulence and potential applications will be reviewed in this work.

## 1. Introduction

*Pseudomonas syringae* populations exist within diverse microbial communities on nearly all terrestrial plant species [1]. This bacterial plant pathogen colonizes the intercellular spaces of leaves and other aerial plant tissues and multiplies by using nutrients from living host cells. This bacterial species is disseminated by water or in association with a plant host and typically establishes an epiphytic population on the plant surface prior to infection of the host. Within the species, there are at least 50 pathovars, which cause a wide range of plant diseases exhibiting diverse symptoms, such as leaf or fruit lesions, cankers, blasts and galls [2,4]. *P. syringae* produces several effectors and virulence factors, such as exopolysaccharides that are involved in the development of chlorosis and necrosis symptoms [5], phytohormones, siderophores, ice nuclei proteins and phytotoxins that can function as virulence factors and contribute to disease symptomatology [6]. Particularly significant is the role of the toxins, and the most-studied toxin produced by *P. syringae* is coronatine, which induces chlorosis [7]. Coronatine is produced by several pathovars of *P. syringae*, including *atropurpurea*, *glycinea*, *maculicola*, *morsprunorum* and *tomato* [8,9]. Coronatine acts as a virulence factor, promotes entry of the bacteria into the plant host by stimulating the opening of stomata [10] and suppresses salicylic acid-dependent host defenses [7,11]. This toxin is composed of coronamic acid (CMA) linked with coronafacic acid (CFA). Due to the close resemblance of CMA and CFA to precursors of the endogenous plant hormones ethylene and jasmonic acid, respectively, coronatine is thought to impact signaling in host plants via the ethylene and jasmonic acid pathways [9]. Another group of well-known toxins are the lipopeptides syringomycin and syringopeptin, which are mostly produced by the pathovar *syringae*. The lipopeptides produced by *P. syringae* are a class of compounds containing a fatty acyl residue ranging from C_5_–C_16_ in length and chains of 7–25 amino acids of which 4–14 form a lactone ring [12]. The combination of a polar peptide head and a lipophilic fatty acid tail is responsible for the amphiphilic properties of these compounds, which can lower surface tension and interact with cellular membranes, thereby altering their integrity [12]. 

Additionally, some pathovars of *P. syringae* have shown the ability to produce small and toxic molecules that act as inhibitors, which have been named antimetabolite toxins, this important type of phytotoxins can interfere with nitrogen metabolism of the host. Their phytotoxic action is usually associated with specific disease symptoms. Chlorosis is the most characteristic symptom of tabtoxin and phaseolotoxin [3,13]; however, mangotoxin seems to increase necrosis symptoms in tomato leaves infected with mangotoxin-producing strains of *P. syringae* pv. *syringae* [14,15]. These antimetabolite toxins are small peptidic molecules that inhibit the biosynthesis of essential amino acids, resulting in an amino acid deficiency [16,17] and interfering in the nitrogen metabolism of the plant host. Nitrogen is a common limiting nutrient for the growth of both plants and pathogens. Successful colonization of plants by pathogens requires an efficient utilization of nutrient resources, including nitrogen, to be present in host tissues. A lack of nitrogen weakens plants, rendering them susceptible to certain pathogens [17]. Antimetabolite toxins have a dramatic effect on host metabolism because they inhibit the biosynthesis of essential amino acids and induce the depletion of the intracellular levels of such compounds, thereby acting as antimetabolites. Some studies suggest that the modification of host metabolism produced by this kind of toxin is focused to the benefit of the pathogen, *P. syringae*. In fact, it has been reported that, during the bacterial infection, there was a change in the pattern of an isoform of glutamine synthetase (GS). Thus, assimilation into glutamine by cytosolic GS induced in response to bacterial infection could be an alternative for nitrogen recycling from infected tissues [18]. Moreover, related studies have shown that tomato leaves infected with *P. syringae* pv. *tomato* contained elevated levels of asparagine along with an increase of glutamine synthetase and asparagine synthetase, suggesting that part of the glutamine could be converted into asparagine for export to healthy parts of the plant to save nitrogen [18,19]. Both glutamine and asparagine are amino acids that are easily assimilated by bacterial pathogens, supporting pathogen growth in infected plants [20]. Therefore, the antimetabolite toxins act on amino acid biosynthesis by inhibiting their corresponding target enzymes, which causes amino acid deficiencies in host cells and the concomitant accumulation of nitrogen-containing intermediates that can be metabolized by the pathogen as nitrogen source [17]. Additionally, the decrease in amino acid levels caused by antimetabolite toxins may also affect protein synthesis in plant cells, thereby hindering important host functions, such as active plant defenses [20]. Therefore, antimetabolite toxins could give toxin-producing bacteria an advantage in adapting to different habitats in competition with other microorganisms and may contribute to higher bacterial epiphytic fitness [15,21,22].

More information on the antimetabolite toxins produced by *P. syringae* pathovars is available from studies of tabtoxin and phaseolotoxin. However, others toxins belonging to this group have been found in different pathovars and inhibiting different target enzymes (Table 1), all of which are present in the urea cycle. In the current review, we tried to present an overview of the body of knowledge available to date on tabtoxin, phaseolotoxin and less well-known antimetabolite toxins such as mangotoxin.

**Table 1:** Antimetabolite toxins produced by different pathovars of *P. syringae.*

Antimetabolite Toxin	Target Enzyme	Toxin-Producing Pathovars	References
Tabtoxin	GS	*tabaci*, *coronafaciens*, *garcae*	[2,23]
Phaseolotoxin	OCT	*phaseolicola*, *actinidae*	[3,4]
Mangotoxin	OAT	*syringae*, *avellanae*	[24,25]
Unknown	Not determined	*tomato*, *apii*	[22,26]
Unknown	Not determined	*aptata*, *atrofaciens*	[22]

This table could be complemented with Figure 4.

## 2. Bioassay for Antimetabolite Toxin Detection

**Figure 1:**
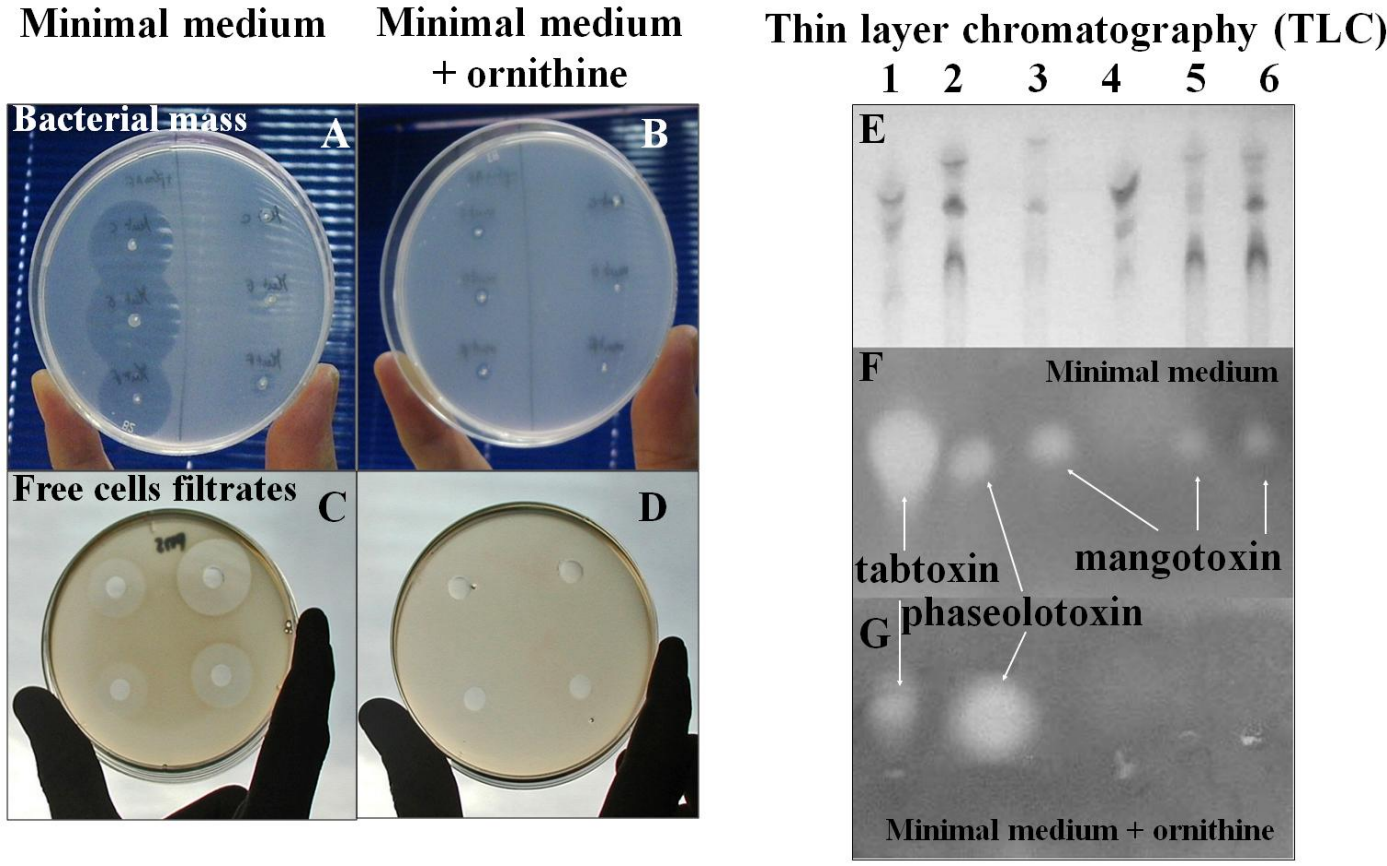
Techniques used to test antimetabolite toxin production by strains of *Pseudomonas syringae* pathovars *in vitro.* Detection bioassay using *Escherichia coli* as an indicator microorganism: (**A**) The bacterial strains to be tested are stabbed into the agar and covered with a thin layer of the indicator microorganism; (**B**) The indicator inhibition can be reversed by one or more amino acids; (**C**) Cell-free filtrates from bacterial cultures can also be used; (**D**) and the toxic activity can be reversed by one or more amino acids. TLC analysis of cell-free culture filtrates of *P. syringae* pv. *coronafaciens* CECT4389 (a tabtoxin-producing strain, lane 1), *P. syringae* pv. *phaseolicola* CECT4490 (a phaseolotoxin-producing strain, lane 2), and *P. syringae* pv. *syringae* UMAF0158, UMAF1003, UMAF2010 (mangotoxin-producing strains, lanes 3, 5 and 6), and the UMAF0158-3αE10 Tn5-mutant (a non-mangotoxin-producing strain, lane 4); (**E**) The fractions were separated by TLC on silica plates, and the chromatograms were visualized under UV light (254 nm); (**F**) The strains’ corresponding toxic activities were located on TLC plates by an *E. coli* growth inhibition assay on a thin layer of PMS agar over the TLC plate or (**G**) PMS supplemented with ornithine.

*In vitro* detection of antimetabolite toxins is mainly based on the indicator technique previously described by Gasson, which involves growth inhibition of *Escherichia coli* on *Pseudomonas* minimal medium (PMS) [27]. In this method, it is important to use minimal medium without amino acids to detect antimetabolite toxin inhibition. Briefly, a double layer of indicator microorganism is made using an exponentially growing strain of *E. coli*. After solidification, the strains of *P.* *syringae* pv. *syringae* to be tested are stabbed, and their plates are incubated at 18–28 °C (the incubation temperature depends on the antimetabolite tested) for 24 h and at 37 °C for an additional 24-h period, if necessary for better observation of inhibition haloes. To assess the biochemical step targeted by the toxin, the same plate bioassay is carried out, but an aliquot of the solution of the corresponding amino acid or intermediate is added to the double layer [24] (Figure 1A,B). A modification of the above techniques can be used to test the toxicity of the bacterial cultures or extracts free of *P. syringae* cells. Different *P. syringae* strains can be grown on liquid media for several days at a temperature adequate for toxin production. After centrifugation, the supernatants are filtered through nitrocellulose membranes. The toxic activity of filtrates can be evaluated by the *E. coli* growth inhibition bioassay that was previously described. To do so, wells are made in the PMS agar plates, and filtrate samples mixed with sterile melted media are placed in each well. Afterwards, a double layer with *E. coli* is added and incubated at 37 °C [24] (Figure 1C,D). Alternatively, paper discs impregnated with the filtrates could be used. In this method, the discs must be placed after double layer with *E. coli* is added [25]. The inhibition zones around the well or discs of visible growth of *E. coli* are measured.

Chromatographic methods also have been used to detect antimetabolite toxins. First, it is necessary to partially purify the toxin from a cell-free filtrate made as described above. An example of the use of these techniques was in the characterization of mangotoxin [14]. The crude cell-free filtrates containing mangotoxin were extracted with an equal volume of methanol:chloroform. The aqueous phases were recovered and concentrated by evaporation *in vacuo*, fractionated on silica gel thin-layer chromatography (TLC) plates and developed in a solvent mixture of methanol:water. The thin-layer chromatograms were visualized under UV light at 254 nm (Figure 1E). Next, the TLC plates were covered with a thin layer of PMS medium amended with 2,3,5 triphenyltetrazolium chloride (TTC) [28], an aliquot of an overnight culture of *E. coli* and the corresponding amino acids to check the specific activities. After incubation at 37 °C for 24 h, areas of growth inhibition of the indicator microorganism appear as haloes with no reddish color, revealing the absence of respiration as a consequence of indicator microorganism growth inhibition [24] (Figure 1F,G).

High-performance liquid chromatography (HPLC) has been used for the purification of antimetabolite toxins, although the method should be adjusted for each antimetabolite toxin because of their different chemical structures. The antimetabolite toxins for which information is available all require a previous extraction or, at least, partial extraction to eliminate molecules that could hinder the purification. Tabtoxin, for example, was extracted from a cell-free filtrate using several columns. The first was a column of Amberlite CG.120, then the supernatant was passed through a column of LH-20 and then the fraction with amino acids was purified by HPLC using a column of Ultrasphere 5-μm ODS [29]. Phaseolotoxin was re-isolated from the culture medium of *P. syringae* pv. *phaseolicola* by charcoal adsorption chromatography, QAE Sephadex ion exchange chromatography and anion exchange (BioRad A-27). Next, reverse-phase HPLC was performed to purify the phaseolotoxin, using the inhibition of ornithine carbamoyltransferase (OCT) assay to monitor the purification [30]. The most recently developed HPLC method for the purification of an antimetabolite toxin was developed for mangotoxin. For the partial purification of mangotoxin, cell-free supernatant fluids were extracted with an equal volume of methanol:chloroform, and the aqueous phases were recovered. After concentration by evaporation *in vacuo* of the aqueous phase, the concentrated samples were injected and fractionated by HPLC with an Alltech Hypersyl ODS 5-mm column. Only one peak showing toxic activity was recovered. It was collected and fractionated by TLC on silica plates. The spot corresponding to mangotoxin was scratched from the plate, extracted and concentrated by evaporation *in vacuo*. To localize the toxic spot, a duplicate TLC plate was developed and used for an assay of *E. coli* growth inhibition in a double layer of PMS-amended TTC, as previously described [24]. 

## 3. Antimetabolite Toxins: Bioactivity, Targets and Role in Virulence

The best-known antimetabolite toxins produced by *P. syringae* pathovars are tabtoxin and phaseolotoxin.

### 3.1. Tabtoxin

**Figure 2:**
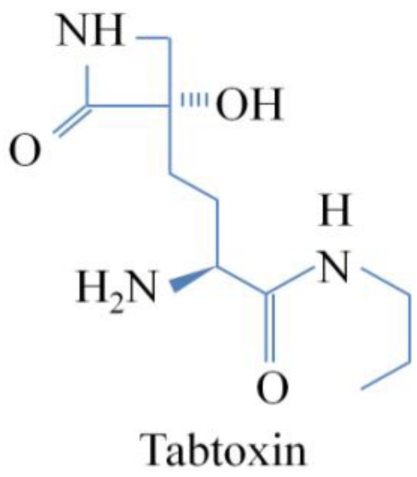
Chemical structure of Tabtoxin.

**Figure 3:**
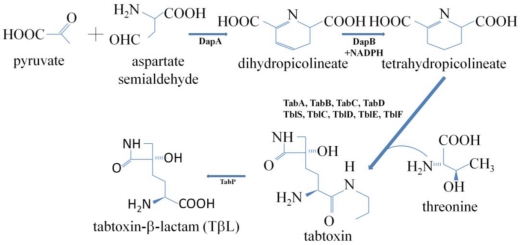
Proposed biosynthetic pathway for tabtoxin. **DapA** dihydropicolineate synthase, **DapB** dihydropicolineate reductase, **TblS** putative β-lactam synthetase, **TblC** putative clavaminic acid synthase, **TblD** putative GNAT acyltransferase, **TblE** + **TblF** putative membrane protein, forming a functional pair with a D-Ala-D-Ala ligase, **TabP** zinc-dependent metallopeptidase. The figure has been adapted from Gross and Loper 2009 [6].

**Figure 4:**
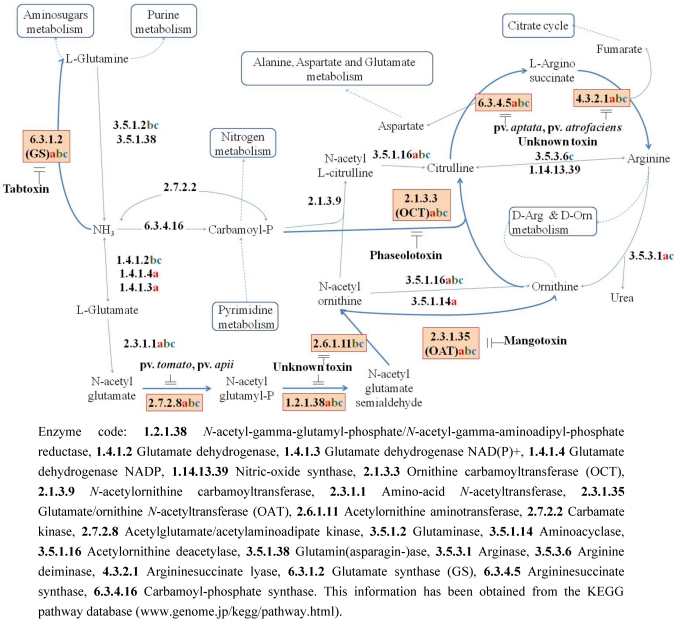
Part of the arginine and proline metabolism scheme obtained from the KEGG website [34], which shows the target enzymes and catabolism steps blocked by the main antimetabolite toxins (shown in orange). The enzymes that are present in plant, whose only representative in arginine and proline metabolism database is *Zea mays* L. are marked (**a**), the enzymes that are present in *Pseudomonas syringae* pv. *phaseolicola* are marked (**b**) and the enzymes that are present in *Pseudomonas syringae* pv. *syringae* are marked (**c**).

Tabtoxin (Figure 2) is produced by *P. syringae* pvs. *tabaci*, *coronafaciens* and *garcae*, and it is the precursor of tabtoxinine-β-lactam (TβL), the biologically active form. The structure of tabtoxin consists of the active form, tabtoxinine-β-lactam (TβL) and serine or threonine [2,23] (Figure 3). The hydrolysis of the toxic moiety, TβL, linked via an amide bond to threonine is carried out by a zinc-activated aminopeptidase present in the periplasm [31]. The physiological target in plants, glutamine synthetase (GS) (EC 6.3.1.2), is irreversibly inhibited by TβL [29] (Figure 4). GS is the main enzyme in nitrogen assimilation in plants, fungi and bacteria. Nitrate, which is the major source of inorganic nitrogen available for plant is, after uptake from soil, either stored in the vacuole or converted into nitrite by nitrate reductase (NR). After conversion, nitrite enters the chloroplast (or cytosol in the root) and is reduced by nitrite reductase (NiR) into ammonia, which is subsequently converted to glutamine by glutamine synthetase (GS) using glutamate as a substrate (Figure 4) [16,32]. The inactivation by TβL is time-dependent, and the rate of inactivation is slowed by the addition of glutamate. Glutamate and TβL compete for the same site on the enzyme. The failure to recover any activity after extensive dialysis of the inactivated enzyme supports the conclusion that the enzyme is irreversibly inactivated [29]. Therefore, TβL irreversibly inhibits GS, resulting in ammonia accumulation, which disrupts the thylakoid membrane of the chloroplast and uncouples photorespiration, leading to chlorosis [16,33].

### 3.2. Phaseolotoxin

**Figure 5:**
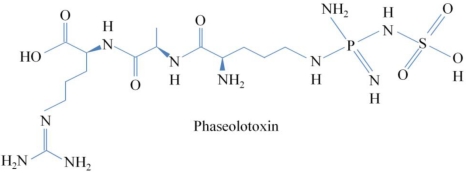
Chemical structure of Phaseolotoxin.

**Figure 6:**
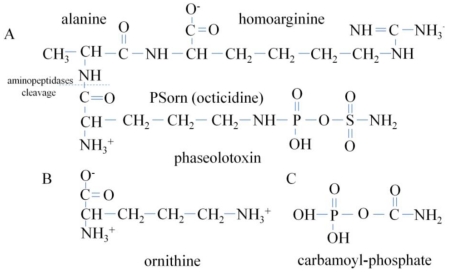
Structures of: (**a**) phaseolotoxin; (**b**) ornithine; and (**c**) carbamoyl phosphate. The figure has been adapted from Templeton *et al*. 1984 [51].

Phaseolotoxin (Figure 5) is produced by *P. syringae* pv. *phaseolicola* and *actinidiae* pathogens, causing halo blight of bean and canker of kiwifruit, respectively, as well as by the *P.* *syringae* pv. *syringae* strain CFBP3388 [35]. Phaseolotoxin is a tripeptide consisting of ornithine, alanine and homoarginine, linked on an inorganic sulphodiaminophosphinyl moiety [3] (Figure 6), and it is an inhibitor of OCT, a key enzyme in the urea cycle that converts ornithine and carbamoyl phosphate to citrulline [6] (Figure 4). The principal symptom produced by phaseolotoxin-producing strains is a chlorotic zone or halo around the necrotic infection site. One of the first studies to determine the relationship between phaseolotoxin synthesis and chlorosis symptoms concluded that chlorosis in the systemically affected leaf was caused by a toxin transported via the phloem [36]. The authors concluded that the downward movement of toxin in the petiole and lower stem may be exclusively through the phloem, while part of the upward movement in the upper stem may occur in the transpiration stream in the xylem as result of subsequent transfer of the toxin from the phloem to the xylem [36]. One decade later, a study about the effects of phaseolotoxin on the synthesis of arginine and protein suggested that one of the characteristic symptoms of plants affected by phaseolotoxin was that growth was stunned when the meristems were affected [37]. This author argued that protein catabolism might be enhanced in phaseolotoxin-treated tissues, as suggested by the findings that net protein accumulation was reduced and that many free amino acids, especially the amides glutamine and asparagine, accumulated in the affected tissue [19,38]. Moreover, the same rate of photosynthesis was found in chlorotic lesions and control tissues. The reason for the reduced chlorophyll synthesis that gives rise to phaseolotoxin-induced chlorosis was not clear, but it appears to involve a reversible block in development, due to a block in *de novo* arginine synthesis at a stage when the young leaf is synthesizing chlorophyll [37].

### 3.3. Mangotoxin

**Figure 7:**
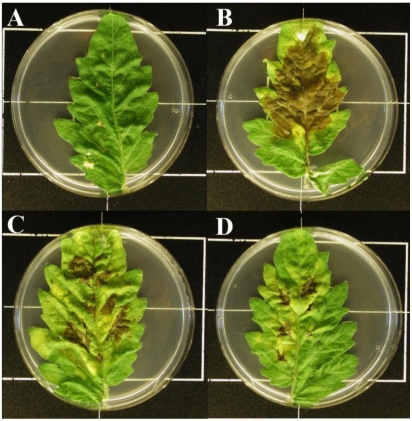
Role of mangotoxin in bacterial virulence. (**A**) Absence of disease symptoms in a control (non-inoculated) leaflet; (**B**) Representative symptoms of a mangotoxin-producing strain of *P. syringae*; (**C**) and (**D**) Symptoms produced by its derivative mutant defective in mangotoxin production on tomato leaflets at 7 days after inoculation.

Least known because of its relatively recent discovery is the antimetabolite toxin named mangotoxin. This toxin is mostly produced by *P.* *syringae* pv. *syringae*, although it has been detected in pv. *avellanae* [25]. Mangotoxin inhibits ornithine acetyltransferase (OAT), which catalyses the synthesis of ornithine from *N*-acetyl ornithine (Figure 4). Its structure remains unknown, but its properties suggest that it is an oligopeptide [24]. In 1983, a short communication by Mitchell and Johnston described a toxin produced by *P. syringae* pv. *syringae* that inhibited the growth of *E. coli* [39]. The large zones of inhibition were prevented by arginine and ornithine but not by triglycine. This toxin mentioned by Mitchell and Johnston [39] could be the same toxin that was later described as mangotoxin by Arrebola *et al*. This toxin was classified as a virulence factor that contributes to the severity of the disease produced by *P. syringae* pv. *syringae*. Therefore, studies to determine the contribution of mangotoxin to the virulence and epiphytic fitness of *P. syringae* pv. *syringae* have been carried out. Mangotoxin-producing *P. syringae* pv. *syringae* strains and derivative non-producing mutants were inoculated in tomato leaflets maintained *in vitro* (Figure 7) All of the assayed strains grew at similar rates and reached similar population densities in and on inoculated tomato leaflets. However, the necrotic symptoms produced on tomato leaflets after *P. syringae* pv. *syringae* inoculation were clearly reduced when the mangotoxin-defective mutants were used, such that mangotoxin was confirmed as a virulence factor [15].

Additionally, most antimetabolite toxins show antimicrobial activity [22], and they could contribute to bacterial competitive ability and epiphytic fitness [21]. Experiments have revealed similar population densities of mangotoxin-producing strains and mangotoxin-defective mutants when they were inoculated individually. However, when the wild type was co-inoculated with mangotoxin-defective mutants, the mutants reached lower population densities. These results suggested that *P. syringae* pv. *syringae* strains producing mangotoxin outcompeted non-producing strains to colonize the phyllosphere [15].

### 3.4. Uncharacterized Antimetabolite Toxins

Other antimetabolite toxins act by inhibiting enzymes associated with the urea cycle (Figure 4). However, it has been impossible to determine which target enzyme is inhibited by either of the producer strains, *P. syringae* pv. *tomato* and pv. *apii.* These toxins could also inhibit an earlier and undetermined step of the arginine biosynthesis pathway, such as acetylglutamate kinase, acetyl-glutamyl-phosphate reductase or acetylornithine aminotransferase. The specific target enzyme of this antimetabolite toxin from *P. syringae* pv. *tomato* and pv. *apii* remains to be determined (Figure 4) [22,26]. Additionally, another antimetabolite toxin produced by *P. syringae* pv. *aptata* and *P. syringae* pv. *atrofaciens* could inhibit enzymes that directly catalyze arginine synthesis because only arginine can reverse the toxin effect [22], but, to date, this enzyme has not been located. Finally, pv. *maculicola* produces a toxin with antimetabolite characteristics; the metabolic inhibition caused by this toxin was not reversed by any of 24 amino acids tested [22]. 

From this section, it is possible to conclude that antimetabolite toxins are compounds produced only in *P. syringae* species; however, their production is not generalized, making it possible to find producing and non-producing strains belonging to the same pathovar of *P.* *syringae* [40]. The antimetabolite toxins act as virulence factors that contribute to pathogen virulence, increasing the disease symptoms, although they are not a determinant of the pathogenesis of *P. syringae*. The known antimetabolite toxins are oligopeptides, and all of them inhibit target enzymes involved in amino acid biosynthesis, interfering with nitrogen metabolism. 

## 4. Chemical Structures of Antimetabolite Toxins

Antimetabolite toxins inhibit target enzymes present in amino acid biosynthesis pathways, therefore, their chemical structures could contain analogues of the regular substrates of these enzymes. 

### 4.1. Tabtoxin

The first studies on antimetabolite toxins were focused on tabtoxin, which has a chemical structure consisting of tabtoxinine-β-lactam [2-amino-4-(3-hydroxy-2-oxo-azacyclobutan-3-yl)-butanoic acid] linked to threonine (and, less often, to serine) (Figure 2 and Figure 3). Interest in this toxin produced by *P. syringae* dates back more than three decades. In 1975, Lee and Rapoport reported the synthesis of tabtoxinine-δ-lactam, an amino acid produced by various *Pseudomonas* species that appears to be one of the compounds found in the hydrolysis of tabtoxin or isotabtoxin. The other hydrolysis products were tabtoxinine and threonine [41]. These authors described tabtoxin as a relatively unstable molecule. At room temperature (25 °C) and pH 7, the biological activity of a solution of the toxin decreases with a half-life of approximately one day, as translactamization occurred to form the more stable and nontoxic δ-lactam isomer, isotabtoxin [41]. Twelve years later, Müller *et al.* published a study on the biosynthesis of tabtoxin, the toxin responsible for wildfire disease, using radioactive precursors. They showed that the molecular structure of tabtoxin consists of L-threonine and the unusual amino acid tabtoxinine-β-lactam, which proved to be difficult to synthesize, due to the toxin’s instability [13]. The biologically active β-lactam tabtoxin readily undergoes intramolecular transacylation on the stable but inactive δ-lactam isotabtoxin. The authors concluded that isotabtoxin appeared as an artifact of the workup, and no effort was made to isolate tabtoxin in its native form because the analysis of isotabtoxin was representative of the biosynthesis investigations [42]. This work established L-threonine as a direct precursor of the threonine moiety of tabtoxin. Therefore, and because the biosynthesis of threonine was well known, the corresponding moiety of tabtoxin could serve as an internal standard for the interpretation of the incorporation of labeled aspartate, glycerol, and acetate into the tabtoxinine moiety. Thus, L-aspartic acid was established as the biogenetic origin of the side chain of tabtoxinine-β-lactam. The question of how β-lactam ring formation might proceed was subsequently raised [42]. A hypothetical mechanism for β-lactam ring closure in the biosynthesis of tabtoxin was formulated according to the mechanism of a similar photoprocess [43]. However, the pathways proposed at that time were based on a chemical reaction point of view. The synthesis pathway of this toxin and its relationship to the lysine pathway was not determined until tabtoxin biosynthesis had been genetically characterized [6]. 

### 4.2. Phaseolotoxin

Phaseolotoxin, like tabtoxin, is composed of several amino acids linked to an inorganic moiety. Phaseolotoxin is a tripeptide consisting of ornithine, alanine and homoarginine linked to an inorganic sulphodiaminophosphinyl moiety (Figure 5 and Figure 6A). Patil reported in 1974 that acid hydrolysis of “phaseotoxin” yielded glycine, serine, valine and two unidentified amino acids [44]. The fraction corresponding to the two unknown amino acids produced chlorosis in bean leaves but, apparently, did not cause the characteristic accumulation of ornithine. In contrast, two years later, Mitchell analyzed a new antimetabolite toxin to which a new trivial name, phaseolotoxin, was given because it was produced by *Pseudomonas phaseolicola* [3]. Phaseolotoxin contained only three amino acids, none of which was apparently contained in “phaseotoxin”, produced symptoms resembling halo blight when applied to bean leaves and was completely characterized as the compound (*N*^δ^-phosphosulphamyl)-ornithyl-alanyl-homoarginine. For these reasons, Mitchell could not assume that the properties of phaseolotoxin were the same as those described in the literature for “phaseotoxin” [3]. This author identified the *N*^δ^-substituted ornithine group as the effective part of phaseolotoxin. However, he observed that there was an additional feature of phaseolotoxin that made it a potential competitive inhibitor of OCT. Moreover, *N*^δ^-phosphosulphamylornithine (PSorn or octicidine [30]) (Figure 6A) was the major product related to detected phaseolotoxin, and it showed the same toxicity as phaseolotoxin in bioassays. This product had a chemical structure in which an analogue of carbamoyl phosphate [3] was attached to ornithine (Figure 6B), potentially accounting for the known effect of bean halo blight toxin on ornithine accumulation and OCT inactivation [45]. OCT condenses ornithine with carbamoyl phosphate to produce citrulline (Figure 3). It might, therefore, be of significance that the substituent group in phaseolotoxin, sulphamoyl phosphate, could be regarded as a simple analogue of carbamoyl phosphate in which >C=O was replaced by >SO_2_ [3] (Figure 6C). More than one decade later, the biosynthetic precursors for the *N*^δ^-(*N*'-sulphodiaminophosphinyl) moiety of phaseolotoxin have still not been identified. Märkisch and Reuter published in 1990 that the homoarginine and ornithine residues of phaseolotoxin were synthesized by a transamination reaction from arginine and lysine. The amidinotransferase showed high substrate specificities for arginine and lysine in phaseolotoxin-producing strains [46].

### 4.3. Mangotoxin

Mangotoxin was detected in the 1990s by the *Escherichia coli* growth inhibition assay, testing *Pseudomonas syringae* pv. *syringae* isolated from mango trees affected by bacterial apical necrosis [26,47]. Preliminary data about the chemical nature of mangotoxin was obtained using toxic cell-free filtrates subjected to different treatments. The toxic activity resisted high temperatures (100 °C) and extreme pH (2 and 12). In contrast, treatments with proteinase K and pronase eliminated the toxic activity. Ultrafiltration tests revealed that mangotoxin had a molecular size of less than 3 kDa and showed a hydrophilic nature after treatment with an organic solvent. These results were compatible with mangotoxin being an oligopeptide. By TLC, the toxic activity from *P. syringae* pv. *syringae*, corresponding to mangotoxin, was associated with a single growth-inhibiting spot with an *Rf*-value of 0.62. This spot was not detected in filtrates from the non-producer mutant. The mangotoxin spot was specifically detected because *E. coli* growth inhibition was reversed with ornithine, whereas the toxicity of tabtoxin and phaseolotoxin remained. Further chromatography analysis by HPLC showed that toxic activity eluted after 10 min was associated with a single peak, which was detected only in filtrates from the wild-type strains and was not present in filtrates from non-producing mutant derivatives [24]. The most recent knowledge about the chemical structure of mangotoxin, obtained using mass spectrometry, suggests that mangotoxin consists of two amino acids linked to a sugar residue [48]. 

In summary, antimetabolite toxins are small oligopeptide molecules of two or three amino acids linked to organic moieties that are directly involved in the inhibition of target enzymes. These molecules or derivatives interfere with the enzyme-substrate bond by forming an irreversible link with the catabolic domain of the target enzyme. 

## 5. Mode of Action of Antimetabolite Toxins

Antimetabolite toxins act as inhibitors of target enzymes, which catalyze the biosynthesis of amino acids that mostly belong to the urea cycle. 

### 5.1. Tabtoxin

Tabtoxinine-β-lactam (TβL) is an irreversible inhibitor of GS; however, GS is also present in *P. syringae* cells. Purified GS from *P. syringae* pv. *tabaci* is irreversibly inhibited *in vitro* by TβL, and the inactivation is slower in the presence of glutamate, therefore suggesting that the inactivation is active-site directed [31]. This enzyme is regulated by an adenylylation system in which the GS is unadenylylated when the culture is growing with nitrate as the sole nitrogen source and is adenylylated when the culture is supplied with ammonia as a nitrogen source. Thus, GS from *P. syringae* pathovars is regulated in a similar manner as other gram-negative bacteria [31,49]. However, the GS of pathovar *tabaci* becomes partially adenylylated when TβL production is initiated. Knight *et al*. conducted a study about the self-protection of *P. syringae* pv. *tabaci* from its TβL. Their results showed that GS from pathovar *tabaci* is only partially adenylylated during TβL production, and a significant amount of GS activity remains available to the bacterium for continued assimilation of ammonia and glutamine production. Investigation of this adenylylation showed that zinc and TβL did not affect the adenylylation state of GS. However, the addition of serine was immediately followed by partial adenylylation of GS in the Tox^+^ strains growing in the tabtoxin-producing mode. These results strongly suggested that the release of serine and, probably, also threonine upon the zinc-activated hydrolysis of tabtoxin causes the partial adenylylation of GS in TβL-producing cultures [31]. Knight also observed that increasing the adenylylation state of GS slowed the rate of the inactivation, such that fully adenylylated GS was completely protected from TβL, and partially adenylylated GS was inactivated very slowly. These finding suggested that adenylylation of GS during toxin production provides partial protection of GS, and the presence of the well-established end-product effectors of GS can increase this protection [31]. However, GS adenylylation is not the only means by which *P. syringae* pv. *tabaci* is protected. Characterisation studies of β-lactamases from *P. syringae* using the strain BR2 identified three different β-lactamases. This kind of enzyme detoxifies TβL by cleaving the β-lactam ring to produce tabtoxinine, an inactive product. Mutants defective in tabtoxin production and tabtoxin-sensitive mutants still produced β-lactamase [50].

### 5.2. Phaseolotoxin

The mode of action of phaseolotoxin on ornithine catabolism was determined by varying the concentration of phaseolotoxin at different concentration of ornithine [51]. Phaseolotoxin appeared to be competing for the carbamoyl phosphate binding site of OCT. The toxin also appeared to be able to bind to the enzyme-carbamoyl phosphate complex. The presence of a transient phase of enzyme activity before a steady state was reached showed that phaseolotoxin shares a characteristic of tight-binding inhibitors in that it had low rates of association with and dissociation from the enzyme [51]. However, co-migration of labeled [^35^S]PSorn and OCT during gel-filtration chromatography provided further evidence of a covalent link between [^35^S]PSorn and OCT, such that 1 mol of PSorn is necessary to inactive 1 mol of OCT [52]. This enzyme is also common in *P. syringae* pv. *phaseolicola*; however, it is insensitive to its own toxin. In 1987, Peet *et al*. published the presence of genes in *P. syringae* pv. *phaseolicola* encoding the two OCTs that differ in their nucleotide sequences, conferring different sensitivities to purified phaseolotoxin. These authors concluded that the genes encoding the phaseolotoxin-insensitive OCT are part of a gene cluster involved in phaseolotoxin production. The close linkage between these genes suggested an explanation for the simultaneous loss of the ability to produce phaseolotoxin and the loss of phaseolotoxin resistance observed in naturally occurring isolates of the pathogen [53].

### 5.3. Mangotoxin

Mangotoxin is the most recent antimetabolite toxin to be detected, and its mode of action is still not well characterized. In 2003, Arrebola *et al.* published the preliminary characterization of an antimetabolite toxin produced by *P. syringae* pv. *syringae* strains that they named mangotoxin because almost all producing strains were isolated from mango trees. The antimetabolite toxin activity that was produced by many *P. syringae* pv. *syringae* strains was not reversed when *N*-acetyl glutamate or *N*-acetyl ornithine were added (as determined with the *E. coli* inhibition assay), but it was completely reversed after ornithine was added, suggesting that mangotoxin targets the catalysis of ornithine from *N*-acetyl ornithine. Therefore, the target enzyme could be either *N*-acetylornithinase (AO, EC: 3.5.1.16) or ornithine *N*-acetyltransferase (OAT, EC: 2.3.1.35) (Figure 4), neither of which had previously been described as target of an antimetabolite toxin. To clarify which enzyme, OA or OAT, was the specific target of mangotoxin, a known inhibitor of OAT, p-chloromercuribenzoic acid (PCMB), was used, and its effects were compared with the inhibition obtained by toxic filtrates from mangotoxin-producing *P. syringae* pv. *syringae* strains. A strong decrease of OAT activity was observed when cell-free filtrates from *P. syringae* pv. *syringae* strains that produce mangotoxin were assayed. The OAT activity decrease was similar to that observed with PCMB. Thus, OAT was established as the target of mangotoxin [24]. OAT is also present in bacteria (Figure 4), but mangotoxin does not seem to affect the bacterial versions of the enzyme. It is possible that mangotoxin-producing bacteria synthesize a mangotoxin-resistant OAT, although the gene that encodes this resistant OAT is still unknown. Moreover, it is also possible that mangotoxin-producing strains perform the OAT catabolic process using alternatives pathways in which OAT is not involved (Figure 4). 

In summary, antimetabolite toxins act as analogues to natural substrates of their respective target enzymes, competing with the catalytic moiety of the enzyme and blocking the enzyme activity. Almost all of the target enzymes except GS, are involved in the urea cycle, and all of them act in the biosynthesis of essential amino acids for protein biosynthesis and nitrogen assimilation.

## 6. Molecular Bases of Antimetabolite Toxin Production

The antimetabolite toxin biosynthetic pathways have been revealed during the identification and characterization of genes and clusters responsible for their production. 

### 6.1. Tabtoxin

In the early 1990s, a chromosomal region from *Pseudomonas syringae* pv. *tabaci* strain BR2 that contained genes for tabtoxin biosynthesis and resistance was cloned [54]. The authors described the biosynthetic region as physically unstable, and spontaneous deletions in this region resulted in both tabtoxin-deficient and tabtoxin-sensitive bacteria and eliminated disease symptoms on bean. Therefore, Kinscherf demonstrated that tabtoxin was required by BR2 for both chlorosis and lesion formation on beans; however, toxin production was not required for growth in plants [54]. Investigations of the genes in this region and their putative functions have shed some light on the synthesis of tabtoxin. Several genes in the cluster showed high sequence identity to genes of the bacterial lysine biosynthesis pathway. Thorough studies have demonstrated that tabtoxin biosynthesis proceeds along the lysine pathway, branching off after tetrahydropicolineate formation and before diaminopimelate formation (Figure 3). Specifically, pyruvate and aspartate semialdehyde are linked by dihydropicolineate synthase, producing dihydropicolineate. Next, the enzyme dihydropicolineate reductase produces tetrahydropicolineate. From this branch point, tabtoxin is assembled by the action of TblS (a putative β-lactam synthetase), TblC (a putative clavaminic acid synthase), TblD (a putative GNAT acyltransferase), and TblE in conjunction with TblF (a putative membrane protein, forming a functional pair with a D-Ala-D-Ala ligase). Upon assembly, the completed tabtoxin can be converted by the metallopeptidase TabP into the toxin TβL (Figure 3), which is subsequently exported by the transporter TblR. The cleavage of tabtoxin occurs only in the presence of zinc, which is required for peptidase activity [6]. Outside of the tabtoxin gene cluster, the gene *ttr* (tabtoxin resistance gene), encodes an acetyltransferase that confer resistance to TβL. Transgene studies indicated that the overexpression of Ttr abolished the chlorotic symptoms caused by either TβL challenge or infection with *P. syringae* pv. *tabaci*. The crystal structure revealed that Ttr is a member of the GCN5-related *N*-acetyltransferase (GNAT) superfamily [55], members of which have been identified as bacterial aminoglycoside acetyltransferases that became resistant to the action of the antibiotics gentamicin and kanamycin [56,57]. The characterization of the Ttr structure suggested that this protein might utilize a single-step catalysis mode to transfer the acetyl group of acetyl-coenzyme A (AcCoA) directly to TβL. Furthermore, histone acetyltransferase (HAT) activity was found, implying an evolutionary link between Ttr and the other members of the GNAT superfamily. He *et al.* proposed a single-step catalytic mechanism as follows: first, despite the large, hydrophobic nature of the putative TβL-binding pocket, the polar environment formed by residues Glu92, Asp130 and Lys95 could assist TβL docking at the active site by adding a tightly bound water molecule. The build-up of negative charge on the oxygen facilitates a nucleophilic attack on the imide carbon of the lactam, disclosing the lone pair on the imide nitrogen of the lactam, which subsequently makes a direct nucleophilic attack on the carbonyl carbon of AcCoA. This carbonyl carbon is polarized by Tyr141, Leu96 and Lys95 through one strong and two alternate hydrogen bonds, respectively. As a result, the C-S bond on AcCoA and C-N bond on the lactam are broken, and TβL is acetylated on the imide nitrogen of the lactam. The negatively charged sulphur of CoA could subsequently be protonated and thus stabilized. Finally, both CoA and the acetylated product leave the catalytic site [55]. Thus, the toxic TβL can be transformed into a non-toxic product.

### 6.2. Phaseolotoxin

Phaseolotoxin biosynthesis is not as clear as that of tabtoxin. From very early in phaseolotoxin study, temperature was determined to regulate phaseolotoxin production. The chlorosis associated with *P. syringae* pv. *phaseolicola* infection on beans was induced at cooler temperatures (18–20 °C) but not at warmer temperatures (28-32 °C) [58]. Subsequent studies showed that phaseolotoxin production decreased progressively at temperatures above 18 °C [59]. Rowley *et al*. observed the production of a repressor when *P. syringae* pv. *phaseolicola* was grown at 28 °C, which is a temperature unfavorable for phaseolotoxin production [60]. Production of this repressor at the nonpermissive temperature could explain the thermoregulation of phaseolotoxin biosynthesis [4]. On the other hand, Zhang and Patil suggested in 1997 that the ORF3 product of the *pht*E locus might catalyze the formation of the ornithine needed for phaseolotoxin production, but biochemical evidence for this function was lacking [4,61]. Several years later, Hernández-Guzman and Álvarez-Morales isolated and characterized the gene encoding an amidinotransferase involved in the biosynthesis of phaseolotoxin [62]. These authors identified the gene *amt*A from *P. syringae* pv. *phaseolicola*, which showed sequence similarity to L-arginine:inosamine-phosphate amidinotransferase. The cysteine, histidine and aspartic acid residues involved in substrate binding were conserved. When Hernández-Guzman and Álvarez-Morales studied *amt*A expression in *P. syringae* pv. *phaseolicola* grown at 18 °C and 28 °C, they observed that the gene expression was thermoregulated, showing significant expression only at the lower temperature and following a pattern similar to that obtained for *arg*K, the gene encoding the phaseolotoxin-resistant OCT (ROCT). This data suggested that they had obtained a fragment from an amidinotransferase gene involved in homoarginine biosynthesis [62]. Actually, the cluster encoding phaseolotoxin production genes (*arg*K-*tox*) contains the gene *arg*K, which encodes the phaseolotoxin-resistant OCT [62,63]. Templeton *et al*. suggested that, although the toxin-insensitive enzyme has significantly lower affinity for carbamoyl phosphate and binds ornithine more slowly than the sensitive enzyme, it should still efficiently catalyze the synthesis of citrulline, provided that the concentration of ornithine is sufficiently high [64]. Peet *et al*. further showed that the OCT-resistant enzyme was not thermosensitive *in vivo* and could presumably support maximal growth rates of *P. syringae* pv. *phaseolicola* at 30 °C. The two enzymes could be maintained by the pathogen to deal with the regulatory constraints imposed by the coordination of phaseolotoxin and arginine biosynthesis [53]. Subsequent studies of OCT-resistant determined that the STRTR binding motif in the carbamoyl phosphate domain was changed to SGRTS in ROCT. In addition, the sequences following the SMG residues in the SMG loop were different. Of the many residues whose side chains form hydrogen bonds with PSorn in the complex with OCT, Gln82 was altered to arginine in ROCT. While the side chain of this arginine, Arg84 in ROCT, could also bind the phosphate group of carbamoyl phosphate, this and other changes to the binding site were sufficient to predict a small but significant difference in the physical and electrostatic topology of the carbamoyl phosphate binding domain. ROCT retained a largely ordered mechanism with carbamoyl phosphate being the first substrate to bind, although there was a random element to the mechanism that was not observed in other OCTs. The higher binding constant for carbamoyl phosphate and conventional inhibitors indicated that the carbamoyl phosphate binding site was altered. Residues thought to be involved in stabilizing the tetrahedral carbon in the transition state were observed in ROCT, however, and this finding suggested that the chemical mechanism of ROCT is also involved in the formation of a tetrahedral intermediate. Structural analysis of the OCT/PSorn complex indicated that PSorn is a strong mimic of the transition state of OCT [65].

### 6.3. Mangotoxin

The synthesis of mangotoxin is currently under study. To detect putative genes involved in mangotoxin production, mangotoxin-defective mutants have been obtained by transposon mutagenesis. Important information has been obtained from several mutants. One of them helped to characterize a gene (*mgo*A) that encodes a non-ribosomal peptide synthetase (NRPS) and is involved in mangotoxin production. Homologues of *mgo*A are present in other *P. syringae* strains, such as pv. *syringae* B728a, pv. *tomato* DC3000 or pv. *phaseolicola* 144A, that fail to produce mangotoxin. This finding suggests the presence of specific genes that are involved in mangotoxin production. In fact, analysis of two Tn5 mutants showed evidence of at least one other gene cluster involved in mangotoxin production and not present in the *P. syringae* pathovars mentioned. One of these two *Tn5*-mutants is disrupted in a gene encoding a putative carboxylase that does not show significant identity with any gene in the sequenced strains of *P. syringae* and seems to be directly involved in mangotoxin biosynthesis [14]. The analysis of the other Tn5-mutants, which were unable to produce mangotoxin, showed disruptions in the *gac*A or *gac*S genes. The GacA/GacS two-component system controls the production of several pathogenicity and virulence factors, ecological fitness, quorum-sensing systems and synthesis of secondary metabolites in different species of *Pseudomonas* [66], and available data suggest that GacA/GacS also regulates antimetabolite toxin production [14,66,67]. 

Therefore, antimetabolite toxins are biosynthesized by non-ribosomal pathways; these toxins are assembled by enzymatic reactions using amino acids and inorganic residues as substrates. The enzymes responsible for antimetabolite toxin biosynthesis are generally encoded in gene clusters that contain genes encoding biosynthetic enzymes that confer self-protection to toxin-producing bacteria. 

## 7. Conclusions and Applications

Antimetabolite toxins are virulence factors that are not exclusive to *P. syringae* pathovars; small molecules with antimetabolite characteristics are produced by other species, such as *Pseudomonas* *aeruginosa*, which inhibits *Bacillus* spp. and *E. coli* [68], and *Rhizobium japonicum*, whose phytotoxins inhibits the growth of *Salmonella typhimurium* [69]. However, in the field of plant pathogens, the production of antimetabolite toxins has not been found outside of the *P. syringae* complex. These toxins are not specific to only one pathovar because the same antimetabolite toxins are produced by several pathovars of *P. syringae*. However, all of these toxins, including the best known as well as those only recently detected and not yet studied, interfere in nitrogen metabolism by acting against target enzymes involved in amino acid biosynthesis pathways. These types of toxins are made through non-ribosomal biosynthesis, and the enzymes responsible for their production are encoded in gene clusters generally located on the bacterial chromosome. These toxins are small molecules composed of two or three amino acids linked to an organic moiety that act as irreversible inhibitors of biosynthetic enzymes of essential amino acids for the plant host. The activities of these antimetabolite toxins on nitrogen metabolism usually promote a disorder in amino acid levels, causing imbalanced levels of intermediates and depletion of the final product. This metabolic imbalance of amino acids leads to chlorosis and even necrosis symptoms in the host plant and probably aids pathogen growth, due to the release of nutrients when cells are affected. To date, only the biosynthesis pathways of glutamine and arginine/ornithine have been identified as target pathways for antimetabolite toxins; however, other biosynthetic pathways are probably affected as well. Therefore, a systematic search for new antimetabolite toxins in *P. syringae* and other phytopathogenic and epiphytic bacteria should be undertaken, especially because the possibilities for the commercial exploitation of new toxins and their corresponding biosynthetic and resistance genes are, in principle, highly promising for the development of new bioactive compounds, resistant plants and biological control agents in postharvest agricultural techniques. To date there are two commercial herbicides that mimic bacterial toxins: glufosinate and bialaphos, which act by inhibiting GS in target plants. Glufosinate is a small compound with the structure of 2-amino-4-(hydroxyl (methyl) phosphonoyl) butanoic acid [70], similar to tabtoxinine-β-lactam, 2-amino-4-(3-hydroxy-2-oxo-azacyclobutan-3-yl)-butanoic acid. Glufosinate inhibits GS by binding to the glutamate site; therefore, plants treated with glufosinate die because of ammonium poisoning and its consequent effect on chloroplasts [70]. Bialaphos is an analogue of tabtoxin and consists of phosphinothricin and two L-alanines [71], and it also acts by inhibiting GS in treated plants, causing an increase in ammonium levels and, therefore, disorganization of the thylakoids. These two herbicides are useful examples of applications of bacterial toxins. Antimetabolite toxins could be an interesting inspiration for the synthesis of stable herbicides based on the inhibition of enzymes involved in the urea cycle.
